# Role of Deep Learning in Predicting Aging-Related Diseases: A Scoping Review

**DOI:** 10.3390/cells10112924

**Published:** 2021-10-28

**Authors:** Jyotsna Talreja Wassan, Huiru Zheng, Haiying Wang

**Affiliations:** 1 Maitreyi College, University of Delhi, New Delhi 110021, India; jtwassan@maitreyi.du.ac.in; 2 School of Computing, Ulster University, Belfast BT15 1ED, UK; hy.wang@ulster.ac.uk

**Keywords:** aging, deep learning, classification, prediction, PRISMA

## Abstract

Aging refers to progressive physiological changes in a cell, an organ, or the whole body of an individual, over time. Aging-related diseases are highly prevalent and could impact an individual’s physical health. Recently, artificial intelligence (AI) methods have been used to predict aging-related diseases and issues, aiding clinical providers in decision-making based on patient’s medical records. Deep learning (DL), as one of the most recent generations of AI technologies, has embraced rapid progress in the early prediction and classification of aging-related issues. In this paper, a scoping review of publications using DL approaches to predict common aging-related diseases (such as age-related macular degeneration, cardiovascular and respiratory diseases, arthritis, Alzheimer’s and lifestyle patterns related to disease progression), was performed. Google Scholar, IEEE and PubMed are used to search DL papers on common aging-related issues published between January 2017 and August 2021. These papers were reviewed, evaluated, and the findings were summarized. Overall, 34 studies met the inclusion criteria. These studies indicate that DL could help clinicians in diagnosing disease at its early stages by mapping diagnostic predictions into observable clinical presentations; and achieving high predictive performance (e.g., more than 90% accurate predictions of diseases in aging).

## 1. Introduction

Aging refers to the persistent decline in the age-specific fitness due to internal physiological changes, anatomical, and immunological changes in living beings [1]. Physiological changes are usually associated with a wide range of disorders, including neurodegenerative, cardiovascular, respiratory, and eye-related diseases [2]. Efforts to early detection of diseases in aging population and related therapeutics have now become a hot topic of research. Cutting edge modern computing technologies in Artificial Intelligence (AI) such as Deep Learning (DL) are being recently applied to improve understanding of aging-related diseased conditions and have been engaged to assist clinicians and healthcare professionals for improved decision-making [3]. DL-based algorithms indicate great potential in extracting features and learning patterns from complex and heterogeneous medical data pertaining to an individual’s health status. Such data may involve medical images, such as scans from imaging devices; genomic data relating human genes to diseases; smart sensor data to detect medical conditions and their effects; data from electronic health records (EHRs); and the time series data from electrograms [4]. DL methods aid in learning these data representations to predict diseased states related to aging [4]. DL transforms the data through layers of nonlinear computational processing units, providing knowledge discovery from the complex data. In recent years, DL algorithms have indicated superior performance in many data-rich application scenarios relating to the healthcare of the aging population.

In previous review studies, Taeho Jo et al. [5] reviewed DL approaches and neuroimaging data for the early detection and diagnostic classification of Alzheimer’s disease (AD), one of the most common diseases in the aging population. Shoukry et al. [6] conducted a mini-review highlighting the use of DL in AD detection. Yue et al. [7] provided a concise review of DL applications in various aspects of genomic research, indicating disease-causing genes. Li et al. [2] conducted a review on aging and age-related diseases. Kieu et al. [8] provided a survey of DL for detecting lung diseases. Wang et al. [9] highlighted the adoption of DL in systems medicine with a special emphasis on predictive modelling for personalized Parkinson’s disease. In their review, Wang and Chen et al. [10] did a survey on using DL in sensor-based activity recognition. To best of our knowledge, a review highlighting using DL in predicting a variety of healthcare issues in aging is missing in the literature.

To extend upon this, we aim to provide a scoping review of the existing research, applying DL methodologies on the analysis of different types of aging-related diseases utilizing different kinds of data to predict diseased conditions. A scoping review is one of the newer review types used to emphasize conceptual boundaries of a topic or field [11]. The boundaries of the current scoping review enclose the applicability of DL models to effectively predict aging diseases and issues in the past few years (2017–2021). This review would help to understand the use of DL in surrogate of age-related health versus diseases with multiple phenotypes, such as biological targets, cardiovascular aging, eye pathology, lifestyle patterns, immune decline, lung function, bone and knee joints aging.

In the current review, we first briefly provide a conceptual framework for applying DL to aging-related diseases with some background on state-of-the-art DL techniques. Then, we perform the scoping review of the literature relevant to DL applications in detecting aging-related diseases. According to the most common aging problems, we categorized the relevant articles from the scoping review into six co-groups. Different types of data were analysed under different diseased states including the following aging-related diseases: age-related macular degeneration, cardiovascular and respiratory diseases, Alzheimer’s, arthritis, lifestyle, and other patterns in disease progression in aging people. We also discuss principal findings and challenges in using DL algorithms to improve diagnostic classification in aging diseases and suggest some promising future directions. 

### 1.1. Conceptual Modelling with DL to Solve Aging-Related Diseases

**Figure 1:**
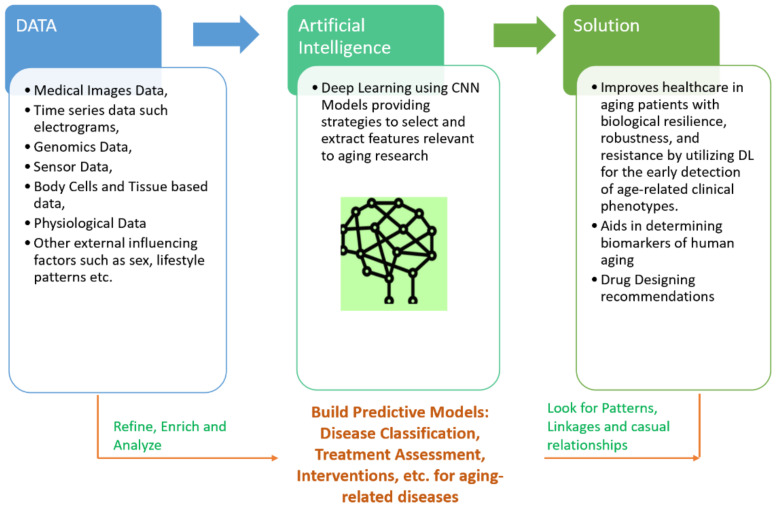
A generalized conceptual framework for applying DL to aging-related diseases.

Aging is a universal mechanism, driven by changes at different stages of life in human beings. In this paper, we review how data from large human medical studies and analytical approaches borrowed from DL can help to fight against aging-related diseases. With such an approach, we hope to generate predictive models for detecting novel therapeutic targets for future aging interventions. The association of most physiologically relevant variables with age is a hallmark of aging studies in humans. Chronic age-associated diseases may share genetic, biological, and psychological traits over a time period. Strategies for human life extension involve targeting resilience with interventions that increase human life, and developing therapies aimed at a reduction in the number of aging-related diseases. DL systems trained on measurable health related variables (changing over time) could help in learning various such biological processes. Combining the prevention and early detection of aging diseases by adding a computational approach of DL would yield better outcomes in that it would promote better treatment and care procedures. Control of the aging process is driven by data acquisition from patients and the control group. Investigations of aging dynamics is supported by experiments with medical images, time series data, and genomic markers, etc. obtained from aging patients and control subjects. DL approaches help perform such investigations and facilitate new clinical trials to minimize functional declines associated with aging. The most common physical health conditions associated with aging are respiratory diseases, cardiovascular diseases, Alzheimer’s disease, arthritis and age-related macular degeneration [12]. We envision the review of joint use of DL over big data obtained from medical images, electronic medical records, research databases, personalized genomics and wearable sensor data for continuous monitoring of aging patients’ health and risks of aforementioned diseases. DL has been successfully applied to imaging data to determine features that are associated with the detection of clinical diseases and also to more accurately classify disease progression in tissue-based studies [13]. DL has also been utilized over blood biochemistry parameters and cell count linked to chronological age with other factors such as sex and lifestyle [14]. DL methods identified albumin, glucose, alkaline phosphatase, urea and erythrocytes as biomarkers for determining the chronological age of human beings [14]. The attractive feature of DL is to identify relevant patterns within complex and nonlinear medical data. A conceptualized framework embedding DL within the complex context of aging-related diseases is shown in Figure 1.

The proposed review aims to showcase the potential of DL-based computational models in clinical and healthcare frameworks for age-related diseases. It focuses on the latest advances in DL approaches to process multi-data sources, such as neuroimages, electrophysiological time-series, multi-modal biomedical data, electronic health records, etc. for deployment in a range of clinical and healthcare solutions developed for aging people, shown in Figure 1.

Remarkably, DL models have achieved clinician-level accuracy at different diagnostic tasks, such as retinopathy and referrals from fundus images [15], optical coherence tomography (OCT) images of the eye [16], and brain analysis with magnetic resonance imaging (MRI) [17].

DL has even been shown to be effective in different medical image modalities (e.g., computed tomography (CT), MRI, ultrasound imaging and planar X-ray, ingle-photon emission computed tomography (SPECT), positron emission tomography (PET) and hybrid imaging systems, such as PET/CT, etc.) [18]. However, real-world clinical settings involve external factors, such as the patient history, health records, any additional tests, such as blood tests, patient testimony, lifestyle patterns, etc., with medical imaging analysis. DL methods also aim to model temporal sequence (time-based sequences) of structured events that were recorded in a patient’s electronic health records with convolutional and recurrent neural networks in order to predict future medical incidents, such as mortality, patients’ stay for medical care and other diagnosis predictions [19,20].

DL has also been adapted to work with genomic data (gene representations) to infer DNA sequences, the effects of DNA mutations on gene splicing, and the effects of genetic mutations on disease risk or drug response. Understanding such genetics of disease allows health care professionals to recommend better treatments and provide more accurate diagnoses [21]. Recently DL has been successfully used to perform the classification of cellular senescence (a state in which human body cells can no longer divide and become a therapeutic target; a hallmark of aging) for finding drugs that control cellular senescence [22].

### 1.2. Background on DL Models 

**Figure 2:**
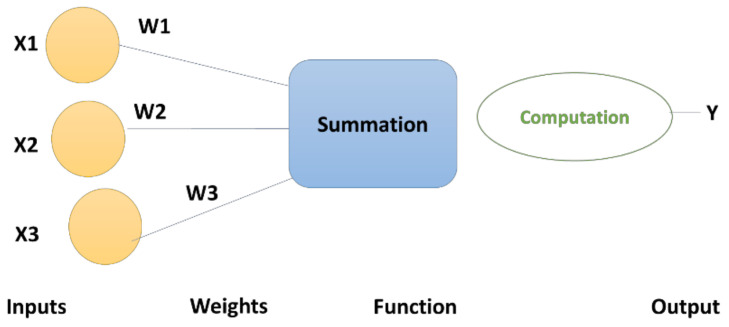
Illustration of an artificial neural network.

DL [23] has emerged as the most important pillar in ML models. It is based on artificial neural networks [24] and is gaining popularity because of its rich applications in the areas of image processing, time series recognition, natural language processing, computational biology, drug designing, and many more. DL is a technique for classifying information through layered neural networks, an imitation of how the human brain works [25]. Artificial neural networks have a set of input units, where raw data are fed. This input can be pictures, sound samples, or written text. The inputs are then mapped to the output nodes, which determine the category to which the input information belongs [25]. Processing in multiple layers in DL refers to a procedure in which the current layer takes the output of the previous layer as an input. The success of DL methods is attributed to their ability to perform feature engineering or transformation layer by layer [26]. Each layer has a functional unit doing the transformation of the data received from the previous layer and then passing the results to the next layer as depicted in Equation (1).

(1) $$m_{k.l}=f\left(x_{1,\;l-1},\;\cdots \;,x_{n,\;l-1}\right)\;\;$$

where *n* is the number of computational units (neurons) in the (*l* − 1)^th^ layer; $m_{k.l}$ represents the output from the *k*^th^ unit in the *l*^th^ layer, and $x_{i,\;l}$ represents the input from the *i*^th^ unit in the (*l* − 1)^th^ layer. Several hidden layers between the input and output nodes, make DL methods capable of making much more complicated classifications of data. Each neuron (also known as a node/cell/unit) is fully connected to nodes in the previous layer by modelling in such a way that the strength of each connection to the previous layer is represented by weight and an activation function, which is applied on the weighted sum to derive output for the next layer, shown in Figure 2.

With the algorithmic development of DL methods, various hardware architectures have emerged in previous years [27]. The trends in DL methods that were recently published are listed in Table 1.

The most famous types of DL methods are discussed in this section; these include DNN, CNN, AE, and RNN [31].

- **DNN**

**Figure 3:**
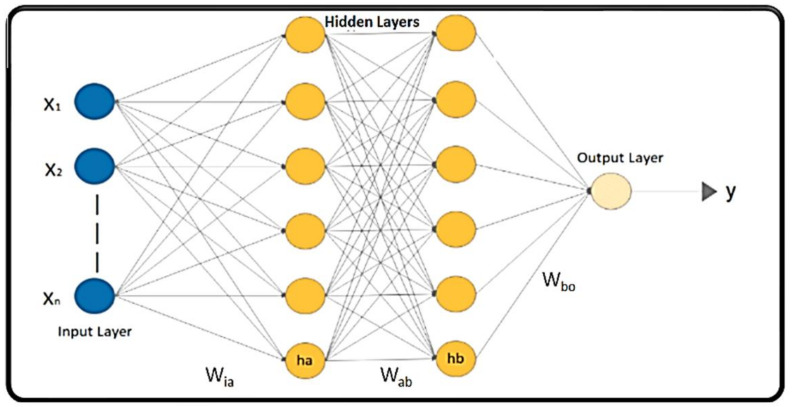
An illustration of a simple DNN with two hidden layers.

ANNs, shown in Figure 3, having more than one hidden layer in their architecture, are known as DNNs, which have the capability to process complex data with the help of mathematical modelling [30,31]. ‘Deep’ in DNN implies the number of processing layers through which the raw data are transformed. DNNs are also known as Feed Forward Neural Networks (FFNNs) as the flow of information is unidirectional in the forward direction. DNNs are DL models composed of multiple nodes (neurons) in a hierarchical organization, inspired by biological neurons of the human brain [28]. The nodes are interconnected with weights on the links to interact with each other in different layers. The nodes have the capability of processing input data and performing simple computational operations on the input data. The result of these operations is passed to other neurons. The propagation function determines the input to the next level neuron from the outputs of its predecessor neurons and their interconnections as a weighted sum. The output at each node is known as the activation (node) value. DNNs are capable of learning, which takes place by altering the weight values [36]. The data are passed from the input layer to the next level layer, until they reach the output layer, where they provide the prediction of *yes* or *no* based on probability. The weights are optimized to make sure that the network makes a correct prediction.

- **CNN**

**Figure 4:**
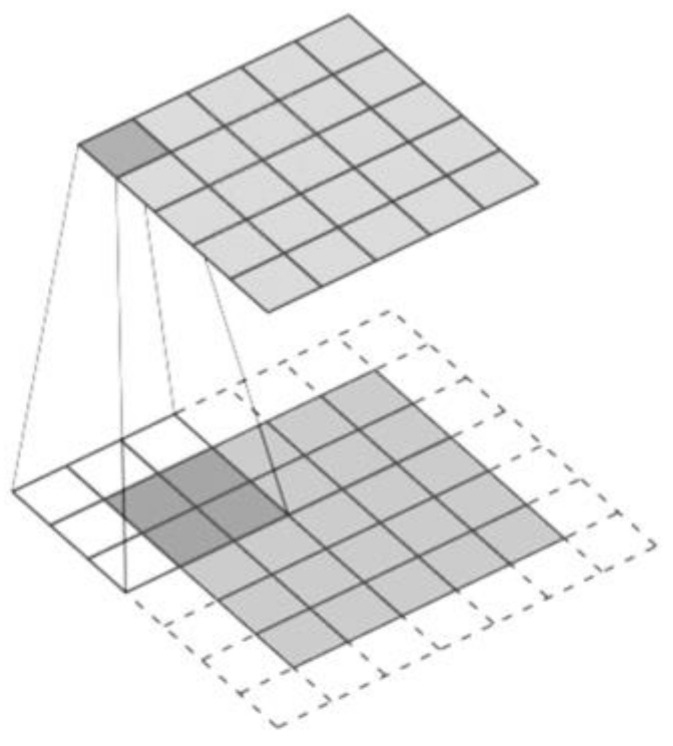
Convolution operation.

The first used CNN was in 1989 for handwritten zip code recognition by LeCun et al. [37]. A typical CNN architecture consists of different convolutional and pooling layers, stacked alternatively [38]. Convolution operation is used to extract features maintaining the spatial arrangement of pixels in input data (primarily images). It involves a linear operation that involves combining an input data with a filter/feature selector (i.e., kernel) generating a feature map depending on three dimensions: depth (number of kernels), stride (the number of data units shifted over the input data) and padding (the number of additional units added to the input), shown in Figure 4 [29,38]. 

The commonly used activation function of rectified linear units (ReLU) is a non-linear operation and is applied to convolved feature maps [39]. Pooling on a rectified feature map reduces the size of each map while preserving the most important features. It slides over the feature map by using famous mathematical operations of max, average and sum functions. Finally, the classification is performed based on the output from the convolutional and pooling layers using a fully connected network. The linear activation function ‘Softmax’ is applied to this last level to derive the final output [39]. CNNs have been primarily applied to image processing, raising the need to carefully transform non-image data, to an image form [5].

- **AE**

AE was introduced in 1986 by Rumelhart et al. [40]. AE is an unsupervised artificial neural network that encodes (compresses) the input data and also learns to reconstruct the original data back from the reduced encoded representation [27]. By using AEs, high-dimensional data can be dimensionally reduced (by an encoder) and reconstructed back by decoder without significant loss of information by learning how to reduce noise in data. A variety of deep autoencoder architectures have been proposed in the literature [31] showing great potential in healthcare.

- **RNN**

**Figure 5:**
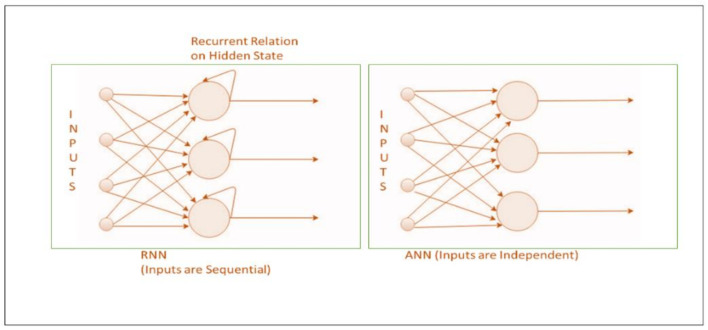
A simple illustration of RNN and ANN.

RNNs process sequential information (inputs) in DL models and retain its previous state while processing the next sequence of inputs [31]. Its architecture is driven by cyclic connection and recurrent units Figure 5. RNN’s hidden state remembers some details on an input sequence. The state at each time *t* is estimated in RNNs based on the previous hidden state and the current input as defined in Equation (2), allowing learning through a recurrent sequential approach [41].

(2) $$h_{t}=f\left(W_{RN}h_{t-1}+W_{IN}x_{t}\right)$$

where $W_{RN}$ is weight at recurring neuron, $W_{IN}$ is weight at input neuron, $h_{t-1}$ is the previous state, $h_{t}$ is the current state and $x_{t}$ is the input state.

These are unlike traditional neural networks which assume each input to be independent of the other Figure 5. A novel RNN approach was recently devised to model dependencies in brain networks based on the mechanism of functional magnetic resonance imaging (fMRI) [5].

- **DBN**

DBNs were introduced in 2007 by Larochelle et al. [42], as a probabilistic generative model that can work better for studies involving traditional neural networks training in deep layered networks, facing issues such as slow learning, poor parameter selection, getting stuck in local minima and requiring many training datasets. Generative models provide joint probability distribution over input data and labels [32]. DBNs consist of several layers of neural networks, also known as “Boltzmann Machines” [34]. In DBNs, training occurs layer-wise while adjusting the weight parameters and balancing the learning probability [31,34]. 

Various DL frameworks are increasingly being used in various kinds of applications. AlexNet was a turning point in the history of DL, providing excellent results in image classification [43]. Other frameworks also subsequently emerged, such as VGGNet, Inception and ResNet for working with ImageNet data [43]. These would be beneficial in their application to medical image data.

### 1.3. Data Types Pertaining to Age-Related Diseases 

**Figure 6:**
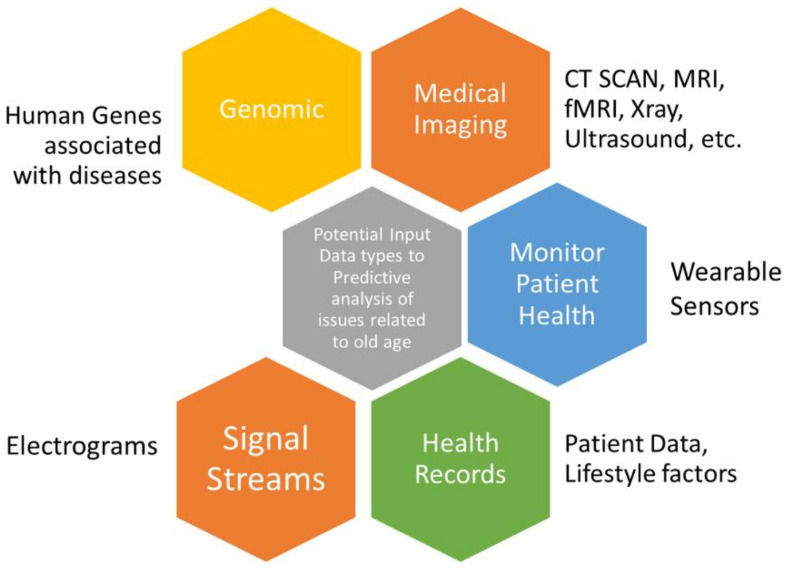
Potential data types in detecting aging-related diseases.

Determinants of age-related diseases may involve a variety of data obtained from varied sources, shown in Figure 6. Variability, quality and complexity of data are the major challenges in analysing such data [44]. Nonetheless, the integration of heterogeneous data types and their use in DL modelling architecture is a potential direction of research [45]. DL facilitates an analysis of the heterogeneous and unstructured data obtained from medical images, medical-grade wearable devices and omics data, with mathematical functions aimed at learning patterns in input data [45,46,47]. Imaging data could involve tomographic imaging modalities, in particular on CT and MRI data, 3D visualizations of the relevant anatomical structures, e.g., 3D ultrasound, positron emission tomography (PET), X-rays, etc. [47].

The revolution in omics approaches (e.g., genomics, metagenomics, and proteomics) has led to a better understanding of human genes and their linkage to diseases. Omics data are becoming more accessible and are capable of classifying genes, as they are associated with phenotypes of multiple age-related [48]. In this context, DL has emerged as a powerful methodology to process probability by which gene associations with age-related diseases are occurring. DL methods provide a supportive layer-by-layer architecture to combine (integrate) several sources of biological data. Human protein coding genes are available from NCBI BioMart v. 87 [49] and age-related disease gene associations can be derived from a subset of the Genetic Association Database [50]. An interesting dataset can be obtained for linking genes with age-related issues from a study by Fabris et al. [48]. Human aging is attributed to a combination of genomic to environmental factors, such as lifestyle patterns, and is henceforth considered to be heterogeneous [2].

Wearable devices have emerged as one of the technological frontiers to associate technology with people’s daily lifestyles for organizing their medication regimen and improving life. Individuals can wear sensors to track various activities, such as cardiac health, treatment effect, physical activity, sleep patterns, potential illness, and disease characteristics [51]. The authors in [52] suggested technology design recommendations for wearables intended for an older population. Wearable technologies help collect data from people to be fed into computational models. Nonetheless, such technology-driven medical and analytical platforms deal with large and complex data which may include high variance and intrinsic collinearity. However, in humans, sometimes because of ethical issues, the capacity of experimentations is limited and, therefore, research related to age-related issues involve observational data, including self-reporting data [53].

One of the most popular applications of DL is to classify image lab test results, imaging and other specialist investigation data obtained from medical care. Image data repositories can be availed from [54]. Integrating electronic health records with OpenEHR, FHIR, and EHCR is at the development stage to provide data management and access for analysis [55].

## 2. Methods

DL approaches have immense potential to improve diagnostics and clinical paths to achieve better outcomes. DL-based approaches offer effective solutions towards data complex medical imaging data processing and analysis, which is useful for the diagnosis of age-related issues [47]. Current methods primarily deal with finding markers of diseases, reporting and detecting human diseases, and monitoring elderly people [56]. For example, DL can involve the heterogenous data of elderly patients [57], use of wearable technology [51] to monitor individual health status, such as cardiac rhythms and movements and their daily lifestyle [58], biometric data [59], and human genes [48], etc., and can advance the analysis of such unstructured data to relevant diagnostic information by removing anomalies and deriving useful patterns. 

DL has been used to detect and classify diseases such as lung cancer and pulmonary nodules from CT imaging [60], neurodegenerative diseases [61], Alzheimer’s disease from fMRI and MRI scans [6], eye-related disorders [15], and detection of skin diseases from photographs [62]. These studies are dependent on large volumes of training data to achieve better DL classification performance. DL approaches, such as CNNs and RNNs, mainly use input data without explicit feature selection and have yielded good accuracies (e.g., 96.0% for Alzheimer’s classification [5]).

DL is a nascent field and has evolved rapidly in the past few years to learn massive amounts of data from cognitive tasks. This review attempts to provide a comprehensive survey of enhancements recently added to the field of applying DL in predicting different age-related diseases. In this paper, we intend to review DL-based models associated with the diagnostic prediction of age-related disease issues. However, one of the key challenges in these studies is the high degree of variability in aging-related changes in response to the cognitive function in people. The predictive and preventive medicinal approach aims to predict the probability of a patient developing a disease, thereby enabling either better medical care by early diagnosis and the treatment of that disease. The use of DL methods has been greatly explored in this approach to identify elderly subjects at high risk for adverse conditions thereby prevention, screening and studying lifestyle interventions.

This review is guided by the preferred conceptual boundaries, as highlighted by Peters et al. in their publication [11]. A Google Scholar, Institute of Electrical and Electronics Engineers (IEEE Xplore), and PubMed search was carried out to identify DL papers on detection of age-related diseases, published between January 2017 and August 2021. These papers were reviewed, evaluated, and classified by different kinds of diseases and types of data, and the findings were summarized. The summary of factors considered in this review is noted in Table 2. 

Although aging issues may occur due to people’s physical and social environments, genetic variations, as well as due to their personal characteristics and lifestyles. These facts may start to influence the process of aging and health at an early stage (40–50 years).

**Figure 7:**
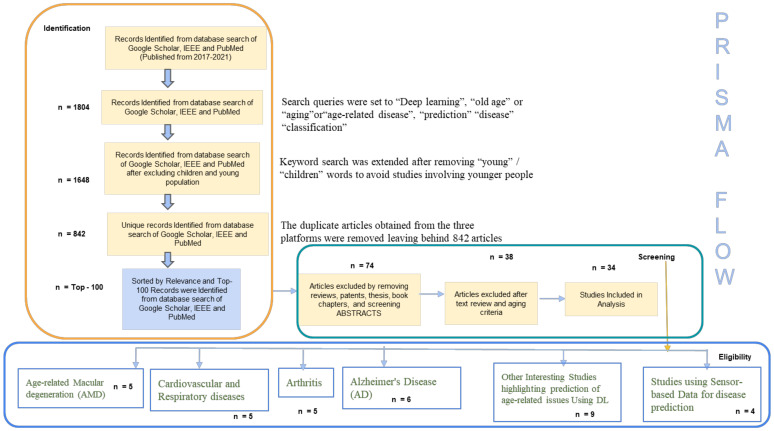
PRISMA flow for the current Scoping Review.

We conducted a review of previous studies that used DL approaches for the diagnostic classification of diseases in people with a mean age of nearly 60–75 years. The search strategy is outlined using the Preferred Reporting Items for Systematic reviews and Meta-Analyses (PRISMA) [63] flow diagram, shown in Figure 7, and is discussed below.

- **Identification**

To thoroughly review the literature, a two-step method was used to retrieve all the studies on a relevant topic—(i) a keyword search was conducted to find relevant articles from Google Scholar, PubMed and IEEE. The keywords used were “Deep Learning”, “old age”, “aging”, “age-related disease”, “prediction”, “classification”; (ii) articles were sorted by relevance.

The sample search queries were set to “Deep Learning”, “old age” or “aging” or “age-related disease”, “prediction”, “disease”, “classification” for Google Scholar; [(Deep Learning) AND ((age-related) OR (old age) OR (aging) AND (disease) AND (prediction) OR (classification))] for PubMed search; and to [((“Full Text and Metadata”: Deep Learning) AND ((“Full text and Metadata”: old age) OR (“Full text and Metadata ”: age-related) OR (Full text and Metadata: aging) AND (“Full Text and Metadata”: prediction) OR-related) AND (“Full Text and Metadata”: Classification) OR -related) AND (“Full Text and Metadata”: disease))].

1804 articles were retrieved by the keyword search on Google Scholar, PubMed, and IEEE. The keyword search was extended after removing “young”/“children” words to avoid studies involving younger people (1648 articles were retained in the process). The duplicate articles obtained from the three platforms were removed, leaving behind 842 articles. The articles were then sorted by relevance via recommendations available at the search platforms listed above. The top 100 recommended articles were considered for the review.

- **Screening**

We excluded survey or review papers, theses, book chapters and patents. To the best of our knowledge, previous studies have conducted a scoping review of DL applications in detecting a particular disease, such as Alzheimer’s [64], lung diseases [8] and mental health issues [65]. However, our scoping review is focused on DL applicability in a variety of common diseases associated with aging, not restricting any of them.

The remaining articles were screened based on the primary focus of the current review. Abstracts were screened to understand the application of DL in aging-related disease prediction. The articles intending to perform diagnostic classification, detection or identifying factors leading to potential age-related diseases were included. Post-abstract, full articles were screened. Articles considering the mean age of patients of 60–75 (nearly) were included in the review. The articles proposing DL, without implementing it, were removed. Preference was given to highly cited articles. In total, 66 papers were excluded after screening.

- **Eligibility**

After the screening step, the remaining 34 papers were included in this review dealing with different kinds of diagnostic classification of the common age-related diseases [66]. These were categorized with keywords into DL analysis related to common diseases of “arthritis”, “age-related macular degeneration (AMD)”, “cardiovascular and respiratory diseases”, “sensor-based lifestyle monitoring”, “Alzheimer’s disease (AD)” with some other aging-related issues involving “type-2 diabetes”, “predicting brain age”, “narrowing of blood arteries”, “COVID-19” effect on old-aged persons, “retinal fundus”, and linking genes to aging diseases.

## 3. Results

**Figure 8:**
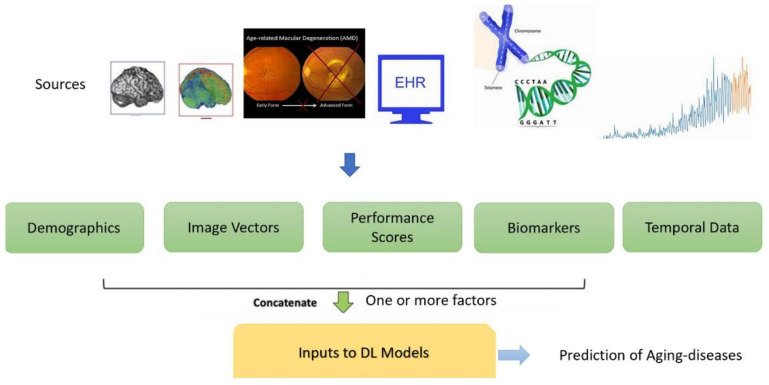
Multimodal feature vectors input to DL methods applied for predicting aging-related diseases.

A total of 34 papers were included in this review. Table A1, Table A2, Table A3, Table A4, Table A5 and Table A6 in Appendix A provides the summary of diagnostic prediction of different age-related diseases with DL-based classification methods adopted in the study. Included articles were examined to retrieve the study of disease condition, application, material and methods with an age range of the population, important findings within the set-up environment and performance of the proposed model. The review deals with DL approaches that could be used to predict varied aging-related diseases with joint use of features, such as images, demographics, medical records, temporal data or genotypes as illustrated in Figure 8. The DL methods play a role in decision support systems for clinicians by increasing the predictive performance. 

This systematic scoping review highlights studies involving various kind of aging-related common issues considering varied input feature vectors (as shown in Figure 8).

- **Age-related macular degeneration (AMD)**

Age-related macular degeneration (AMD) is one of the leading causes of visual loss in the aging population (60–90 years). Methods of DL are useful in better predicting the severity of AMD using imaging methods. DL methods could be used for the public screening or monitoring of AMD worldwide and could further assist in referring the aging population, susceptible to AMD, to a health care provider.

In an interesting study by Qi Yan et al. [15] examined genotype and fundus images of AMD patients which were used as inputs to DL models to dynamically predict an eye that is progressing to the late AMD state, providing disease-severity-related phenotypes. The study indicated that AMD is associated with age, smoking status and a number of genetic variants. As late AMD is irreversible, such a prediction in the early stages would aid patients to adopt preventative care, slowing disease progression. DL methods served as efficient decision support systems (averaged area under the curve (AUC) value of 0.85 (95%CI: 0.83–0.86)), thereby providing various eye services by reducing assessment time and finances via automated analysis. The authors provided a web-based application available online: http://www.pitt.edu/~qiy17/amdprediction.html (accessed on 17 October 2021) to predict AMD state, using both fundus images and genotypes. This could better aid clinicians in predicting year-wise progression of AMD and providing preventive care for patients. The study by Chuan and Yeung et al. [67] served as the first study to use multimodal DL–based architecture for detecting multiple retinal vascular vision threatening eye diseases using multiple image modalities, including retinal fundus photography, optical coherence tomography (OCT), and fluorescein angiography with or without indocyanine green angiography (FA/ICGA). AUCs of 0.987 and 0.969 were attained for predicting retinal vascular diseases and for predicting eye-treatment-requiring diseases. Multimodal imaging is similar to real-world ophthalmology practice, helping in the early screening of the eye diseases and treatment requirements, saving time and making it easy for ophthalmologist on reviewing the images. 

Peng et al. [68] proposed a DL-based model “DeepSeeNet”, which closely resembled the decision support system of clinicians to identify the severity of patient-level AMD using fundus images of both eyes. It is not purely a black-box approach and rather simulates the human grading process by first detecting individual risk factors, such as pigmentary abnormalities in each eye and then combining values from both eyes to develop a disease score for the patient. DeepSeeNet performed patient-based AMD severity classification with a higher level of accuracy than as predicted by a group of human retinal specialists.

Burlina et al. [69], proposed that DL-based models could help in better determining the 5-year risk of progression to advanced stages of AMD. DL approaches support automated and more granular classification from fundus images, unlike traditional computational models, depending on computing or human engineered features from eye-image classification. In the study, the authors created a 9-step scale supporting the more detailed predictability of the potential development of advanced AMD in human beings. In this process, DL-based methods refined retinal diagnostics by delineating fundus and OCT images. The authors highlighted that the DL-based approach allows for better predictive accuracy even in the absence of trained human experts. This could help in the identification of patients at very high risk of progression to advanced AMD. Such patients could be given better counselling for taking preventive treatments at the initial stages. 

Banerjee et al. [16], proposed a hybrid sequential prediction model, called “Deep Sequence”, integrating engineered imaging features and demographic factors to predict the risk of developing non-exudative AMD eyes. Spectral-domain OCT (SD-OCT) represents a gold standard in diagnostic imaging and the management of macular diseases. DL was used in this study to determine indicators of possible disease progression from SD-OCT scans collected at different timestamps (from 3 months to 21 months) along with the demographic features. Imaging features extracted from SD-OCT describing the presence, number, extent, density and relative reflectivity of drusen were used as predictors of AMD progression.

With the advent of more advanced-feature extraction and classification, using DL seems to be more supportive of the clinical assessment in early intervention studies to identify aging patients with high risk for progression to advanced AMD. Careful monitoring and detecting preferred practice patterns, identifying individuals at the intermediate AMD stage in a timely manner, can reduce the risk of vision loss due to AMD in aging persons. DL aids in the fine categorization of clinically relevant features of AMD to guide patients who need an ophthalmologist’s opinion. Table A1 in Appendix A summarizes interesting studies relating DL to AMD.

- **Cardiovascular and Respiratory Disorders in Aging People**

The prevalence of cardiovascular and respiratory diseases also increases with age and could be the cause of morbidity and mortality in older patients. Table A2 presents a summary of studies relating to cardiovascular and respiratory diseases in the scope of the current review. Heart disease is the first cause of death after age 65 [70]. Henceforth it becomes important to deal with such issues with advanced methods.

In a study by Zhang et al. [70], DL techniques were applied to predict complications of coronary heart disease in aging patients with an accuracy rate of 87.50%, which further provided a guiding nursing plan. High-risk patients with coronary heart disease related to old age, medical history and lifestyle patterns to predict complications for implementing a better care plan.

The authors in [71] trained DL models on heart MRI videos, ECGs and heart health indicators obtained from UK Biobank participants to identify biomarkers and clinical phenotypes, associated with accelerated heart aging. Heart aging is a measure of the changes that have accumulated in the individual’s heart over their life span having two main heart facets of heart anatomical (MRI-based) and electrical (ECG-based) aging. These facets contain valuable signals and information which needs to be processed at a pace surpassing “traditional” analytical methods. Using DL methods provided a platform to integrate, analyse and make predictions based on the heterogeneous data of MRI scans, ECG signals and environmental phenotypes of age, smoking and hereditary status. DL was used in [72] to analyse chest radiographs for obstructive lung disease in aging people. The results in [72] indicate that a DL Image Model, improves the detection of obstructive lung disease compared to current practices. The results can be used to direct patients to the medical care of pulmonary diseases and lung cancer screening augmenting radiology clinical reports.

The authors in [73] further studied 10 common abnormalities on chest radiographs in aging people, namely (pneumothorax, mediastinal widening, pneumoperitoneum, nodule/mass, consolidation, pleural effusion, linear atelectasis, fibrosis, calcification and cardiomegaly) using DL models to establish their diagnostic accuracy and timeliness of reporting. The proposed approaches rearranged chest radiographs improving radiologists’ performance, shortening the reporting time for critical and urgent cases.

In an interesting study [74], DL was applied over electronic health records to determine the top 20 factors related to lung cancer instead of prediction relying on self-reported parameters, such as smoking, family, socioeconomic status, or BMI history of patients. Time-related sequential information was also used as a parameter to evaluate lung cancer risk. The use of a such predictive model could serve better to limit unnecessary radiation exposure as well as to reduce the cost.

- **Aging People and Arthritis**

The progression of arthritis in the subjects, reported in Table A3 in Appendix A, indicates interesting studies on how DL could be useful in solving arthritis-related issues in aging subjects. The studies have shown that DL can be used effectively to prognosticate joint pain or arthritis outcomes. Such diseases could otherwise trigger inflammation that could lead to irreversible damage in aging people. The study in [75] demonstrated a comprehensive classification and regression analysis using a novel DL on rheumatoid arthritis to determine concrete numerical predictions of disease activity instead of just classifying high or low risk patients, henceforth making treating-to-(predicted)-target strategies better. It was observed that female patients face a higher risk of clinical progression in rheumatoid arthritis. Potentially, lifestyle, sleep or nutrition also contribute to disease prediction. The DL model developed serves as a potential tool for clinical decision support for patients suffering from rheumatoid arthritis. Leung et al. [76], predicted the risk of osteoarthritis and the likelihood of the patient undergoing the total knee replacement, using DL models. These models accurately predicted osteoarthritis progression in patients requiring a total knee replacement within a nine-year time span than traditional grading systems [76]. In this prognostic study [77], electronic health records were monitored for medications, patient demographics, laboratories visited, and of disease activity measures using DL models to prognosticate future patient outcomes for rheumatoid arthritis. This study forecasted RA disease activity for future clinic visits to better guide specialized treatment on an individualized basis. DL methods measured RA disease activity scores across two healthcare systems and suggested that the disease activity, laboratory values, and medications combined together are the strongest predictor of RA at every clinical visit. DL models trained on the large and diverse patient populations proved to be robust and provided useful insights for patient care. In an interesting study by Hirano et al. [78] applied DL methods to assess radiographic finger joint destruction in RA by analysing images of proximal interphalangeal (PIP) and metacarpophalangeal (MCP) joints. The performance of the model was compared with the scores assigned by both the model and clinicians (rheumatologists). Image processing with the DL model showed promising results to assess radiographs in RA. Assessment by the model was observed to be <1 s per image which proved faster than humans to make an assessment.

- **Alzheimer’s Disease (AD): A Common Disease in Aging People**

Alzheimer’s disease (AD) is a progressive brain disorder that gradually destroys brain memory, it is a common disease in aging people, which is caused by dementia. DL approaches have shown promising results for automated diagnosis and the multi-class classification of AD using resonance imaging and tomographic images (Table A4 in Appendix A). Table A4 highlights the review of studies applying DL over AD subjects. The accurate diagnosis of AD is important, especially at the disease’s early stages, so that patients undergo preventive measures even before the occurrence of irreversible brain damage. Deep Learning (DL) has become a common technique for the early diagnosis of AD. Brain imaging techniques are used to visualize the structure and function of the human brain. The most commonly used imaging technique of MRI helps in measuring brain volumes indicating any kind of degeneration due to AD. For the functional connectivity studies of the human brain, independent components analysis (ICA) has been widely used for analysing neuroimaging data [79]. In the study by Qiao et al. [80], a DL-based method was developed to distinguish AD from controls by fusing the functional connectivity. The study detected the underlying biomarkers of AD by analysing functional MRI. Intrinsic functional connectivity in AD patients was noted to be significantly reduced in subcortical brain regions of the hippocampus, amygdala, insula and putam [80]. 

In a study by Qureshi et al. [81], AD patients with a clinical dementia rating (CDR) were considered, and DL methods applied to functional magnetic resonance imaging data of CDR indicated that the medial frontal, sensorimotor, executive control, dorsal attention, and visual-related networks mainly correlated with dementia severity. The automatic classification of AD severity groups has important contributions to clinical practice. The study suggests that the DL-based classifier acts as a severity indicator, objectively and accurately complementing the CDR scale in the evaluation of AD severity in the absence of trained neurologists. This could further help in drug treatment according to the stage and symptoms of AD.

Choi et al. [82], developed a brain image interpretation system, based on DL to accurately predict cognitive decline in mild cognitive impairment (MCI) patients using fluorodeoxyglucose and florbetapir positron emission tomography (PET) images. The authors claimed their main contributions as to apply a DL model trained for differentiating AD from controls, and to achieve the accurate prediction of cognitive decline with minimally processed multimodal neuroimage data. Ding et al. [83] also developed a DL method to improve the diagnosis accuracy of AD from fluorine 18 fluorodeoxyglucose PET of the brain. The study [83] analysed whether or not the PET image belongs to class AD, MCI, and non-AD/ MCI. The proposed method [83] could be integrated into clinical workflow and serve as an important decision support tool to aid radiologists and clinicians with early prediction of AD from PET imaging studies. On another hand, the study in [84], highlighted that the invasive nature of PET-based processing and the low sensitivity of identified biomarkers in PET-based studies may affect their application in real world routine clinical settings. Henceforth, the study in [84] aimed at building an AD diagnostic classifier using deep learning/transfer learning, based on brain imaging-based MRI data obtained from more than 217 different brain sites, constituting the largest sample. The researchers [84] applied a DL-based model to build a sex classifier in transfer learning for the objective diagnosis of AD. Nonetheless, the study [85] proposed a multi-modality AD classifier taking into account both MRI and PET images of the brain areas as the inputs, and provided predictions in classifying AD vs. controls, facilitating a fast-preclinical diagnosis. It highlighted that the combination of two types of modality imaging data generates better results [85].

- **Prediction over Spectrum of Age-Related Issues**

The studies [17,19,86,87,88,89,90,91,92,93,94,95], highlighted DL-based prediction of other age-related other issues such as Type -2 diabetics, COVID-19 in older patients, coronary blockage in arteries, age-related eye diseases, brain age with old age, age-related disease gene associations, and heart stroke. Interesting findings and summary of these studies [17,19,86,87,88,89,90,91,92,93,94,95] is provided in Table A5 and Table A6 in Appendix A.

**Figure 9:**
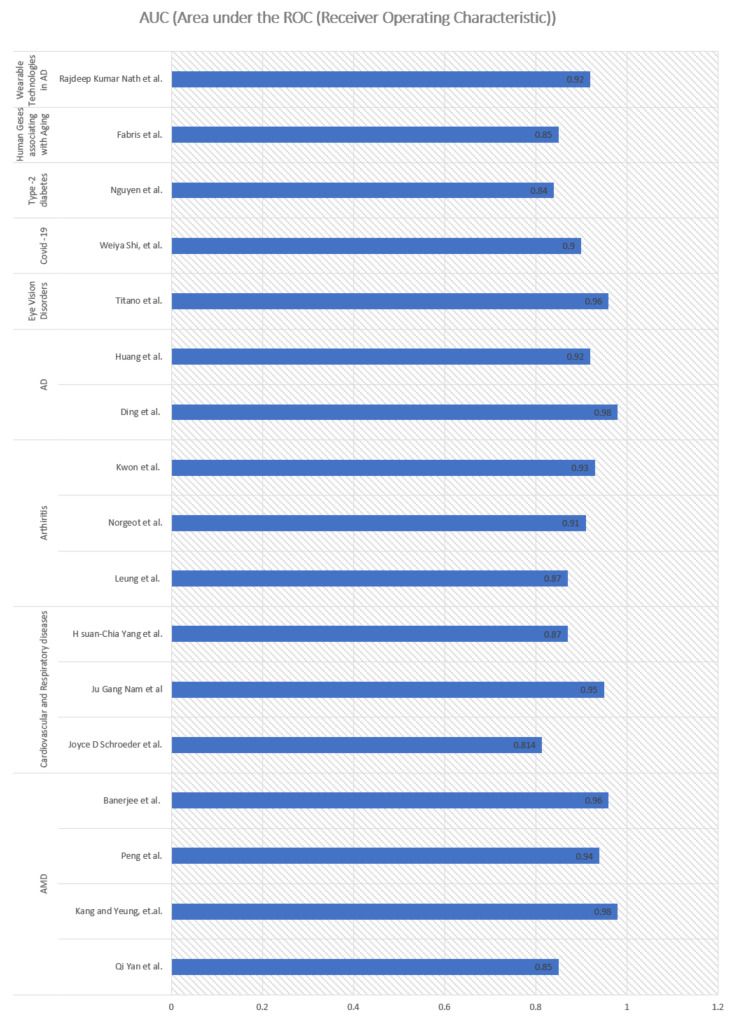
Predictive performance of DL methods applied to aging-related diseases.

Shown in Table A1, Table A2, Table A3, Table A4, Table A5 and Table A6 are the AUC measuring the area under the ROC (receiver operating characteristic), a trade-off curve between the true-positive-rate and the false-positive-rate [96]); accuracy, measuring the fraction of predictions that the proposed model got right) [96] and mean absolute error, which is a measure of errors between paired observations expressing the same phenomenon [97], all of which are noted as the performance measure of the reviewed publications to indicate their effectiveness. Better AUC and accuracy indicate better predictions, whereas lower MAE indicates better predictions. In general, overall high performance was achieved (>84% AUC) by adopting DL models in predicting aging-related diseases (Figure 9). Remarkable performance (high AUC of 98%) was achieved in studies involving DL [83] to predict AD in aging people.

The studies (Table A1, Table A2, Table A3, Table A4, Table A5 and Table A6 in Appendix A) have demonstrated that the use of DL techniques in analysing medical images, such as frontal and lateral images of the heart, MRI, chest scan images, neuroimages, and fundus images, provide evidence in terms of predicting different age-related issues, which could further facilitate their diagnosis. However, the imaging data typically lie in a high-dimensional space which may lead to the risk of overfitting by the DL models. To address this, feature engineering tools are needed, especially in the case of small sample size and high dimensionality. As reported in some selected studies [16,80,90,92,98], DL models can benefit from their feature engineering capability and have been shown to outperform traditional ML models in the prediction of multiple age-related conditions. CNN has also been used to extract features from input ECG signals to predict cardiovascular diseases [71]. ECG data are primarily streaming data that are continuous and are of high density. The studies indicated that the DL model can capture the spectral changes in ECG well to distinguish different states (diseased or not). For EHRs, it was observed that longitudinal DL models performed better [77]. 

The use of genomic data in DL in studying age-related conditions shows promising direction, but domain knowledge is needed to guide the DL model extracting patterns [48]. Some interesting studies have shown the usage of sensor data for guiding and predicting age-related issues [87,88,95]. However, data gathered from sensors may have a certain degree of errors, noise, or redundant information due to battery or communication loss in sensor readings. Hence, such studies prefer to use pre-processing steps such as denoising, transformation, or segmentation for dealing with noise before the application of DL methods.

## 4. Discussion

DL has been playing an important role in providing healthcare professionals with insights, which aids in the detection of health issues early on aging, leading to better patient care. DL has been used in various spheres involving medical image analysis of critical aging diseases, genomics to link genes with diseases, EHR data for personalized care, analysing medical history and providing drugs based on it, cell scope recording medical data on devices, and decreasing frequent visits to consult clinicians. Different types of un-structured and complex data emerging in today’s medical world around aging people are converted into useful formats using DL models. DL models further aid clinicians in the medical classification of diseases, medical resilience, segmentation, cellular senescence, and various other tasks. DL is recommended in dealing with the health of elderly people due to its following benefits:

- DL learns the important patterns or relationships in large amounts of healthcare data and allows clinicians to perform model-based analysis integrated with their observations; leading to smart care achievable from such big data.
- Remarkably, DL has achieved human-level performance in disease classification, learning over patterns/objects contained in medical images.
- When DL is applied over the training data, it becomes more precise with multi-stream architecture and subsequently provides more accurate insights into care processes and diagnostics of aging diseases.
- DL helps in the detection of clinically relevant features by learning patterns in medical imaging data beyond as perceived by a human observer/clinician.
- DL approaches are now leading to lower costs and improved and faster outcomes in monitoring the health of aging people.
- DL provides end-to-end learning models for heterogenous, uncertain and complex medical data.
- DL provides clinicians with the support they need to understand medical environments.

The purpose of this review is to investigate state-of-the-art applications of DL in studying and predicting diseases that may relate to aging people. Out of 1804 articles on Google Scholar, PubMed and IEEE, based on our keyword-search terms and subsequently sorting by relevance, 34 studies met our inclusion criteria, and these were reviewed. Interestingly, these involved varied data types, such as imaging data [78,81,82,89,90,91,98], EHR data [77], ECG data [71], fundus images [67,69,92] and sensor data [87,88,95]. From these studies, we observed that there is a surge in the application of DL methods for studying such diseases. Multiple studies have developed DL methods for disease prediction using clinical and/or non-clinical data and attained promising results. Compared with the conventional classification ML models, most of the reviewed studies involving DL models reported higher prediction performance [72,75,86,88]. These findings pave the path to DL models, assisting clinical experts in better diagnosing health conditions in elderly people. 

By making accurate automated diagnostics of a variety of aging-related diseases, DL methods help in preventing reporting delays for critical conditions, reducing the burden on clinicians, and decreasing errors in diagnosis by auditing diagnostics and care. DL models can make effective interpretations by flagging important areas in medical-related images of aging people, such as in early detection of AD, diabetic retinopathy, and lung nodules. DL-based modelling also applies its neural network architecture to electronic patients’ records, medical reports, time series of electrograms, insurance records, etc. to provide the best outcomes. Thus, DL provides patients with better treatment.

DL is also used to understand a genome and help patients to identify genes causing diseases that might affect them [48]. Nowadays, patients can monitor their health with smart sensor-based devices in real time, thus minimizing frequent visits to the doctor. DL over data collected from smart devices could aid patients in providing better health care [87].

In this review, we collected studies that analyse different types of clinical information, highlighting its ability to address disease predictions in aging people. With the advent of omics sciences, it is imperative to take an integrative approach combining multi-omics data along with clinical information, deriving useful cellular functions to understand age-related diseases more systematically and holistically. DL models seem promising in the area of integrating domain knowledge to identify meaningful patterns from medical profiles [15,71]. However, experts fear that, due to the design architecture, DL models appear to be “black boxes”, without showing details of inner working to reach from raw images for disease predictions [99,100]. Nevertheless, efforts have been made in improving the interpretability of DL in healthcare [101]. Three essential components in DL models are: the learning goal, objective function (or loss function) to be maximized or minimized; and a set of learning rules for networking architecture frameworks as discussed in Section 1.2. In this framework, researchers are not concerned with how computations are performed, but rather enable the learning of computations with the three above-mentioned components (goal, objective-function and rules) [101]. However, DL models developed for neuroscience and healthcare could benefit from better optimization of objective functions with the constraints handling diverse biological and medical data.

It is expected that DL black boxes would yield better impact when combined with predictive modelling approaches where existing medical domain expertise is formally integrated, generating better and novel treatments, as highlighted by Geerts et al. [102]. Additionally, the study in [82], created an interpretation system, based on a deep CNN, to predict future cognitive decline in Alzheimer’s disease. Delivering clinical impact is one of the key challenges for applying DL in the field of healthcare predicting human diseases. As highlighted in this review, applicable DL systems [82,88,94] have been developed; however, it has been argued by the researchers that putting DL technologies from research into clinical practice requires robust system design and clinical evaluation [100]. The format of presentation is an important criterion, while considering the DL-based disease prediction model in clinical practice [103]. A prototype model should undergo a detailed validation process before it is deemed suitable for clinical use [103].

In the disease prediction estimation of the probability that a patient suffers from a disease or not, clinicians would be interested in recognizing patterns in data associated with the disease for guiding better interventions. Rapid progress in imaging and sequencing techniques has made it challenging to integrate large-scale, high dimensional multimodal medical data. Therefore, the research community is progressing towards the use of DL approaches for integrative analysis.

Nonetheless, one of the challenges in the field of applying DL to the medical domain is the availability of large-scale data pertaining to patients. This could lead to the problem of the “curse of dimensionality” which demands the careful optimization of the model parameters to avoid overfitting. Many modelling parameters tend to adapt a DL model too much, leading to overfitting. However, this could be reduced through various regularization methods in DL [104]. DL algorithms usually assume sufficient and balanced training data, which may also not be possible in some real case healthcare scenarios.

Most studies pertaining to the application of DL in disease detection uses features automatically extracted by DL models, such as CNN. However, as one of the future pointers, an ensemble of several features may be explored to provide better detection results. An ensemble of different DL methods could also be considered for potentially better predictions, because ensembles perform many times better than a base classifier. One potential way to reduce the dimensionality of the data is by feature engineering before feeding information to the DL models or the use of transfer learning [85,91]. The transfer scheme was demonstrated to be able to improve predictive performance.

Additionally, due to ethical issues involved in human studies, some researchers used personal datasets. However, more public repositories could be built after deidentification as it would provide the research community with more data to test the efficacy of DL models. Another limitation that has been observed is that the DL models become computationally expensive to train images of larger size, and sometimes it is also time consuming to train a DL model over such big data. 

The studies (Table A6) involving data collected from sensors for aiding health care and lifestyle monitoring in old age, may have a certain degree of erroneous, or noisy information because of the involvement of sensors, which poses a challenge to form computational modelling. Henceforth, pre-processing steps (e.g., data denoising, data and transformation) are necessary before inputting the DL models. 

Due to characteristics of data involved in the medical care of heterogeneity, longitudinal, patient-centred records (both structured and unstructured), and streaming of data (e.g., electrograms), analyses are computationally more expensive, which poses a challenge for the DL model architecture selection. An efficient model should aim for fewer training parameters. Due to domain heterogeneity, researchers have the chance to study age-related problems from different aspects, such as genomic, clinical, medical imaging and sensor signals. Integrative modelling of such multimodal data could provide better insights into elderly diseases. 

The “No Free Lunch Theorem” indicates that no learning algorithm can perform well on all possible issues/problems [105]. Hence, no one DL model could be chosen to perform better in diseased use cases.

Future pointers indicate enhancing DL techniques with the therapeutic intervention of diseases. To discover complex disease patterns with different facets from medical data, computational models need to go deep and various DL architectures (with careful modelling of parameters), hold great promise in this endeavour. Healthy aging is becoming important and DL architectures hold the promise for addressing the challenges of old age.

## 5. Conclusions

The current scoping review focused on the use of DL techniques to study the diagnosis and prognosis of aging diseases in the growing population. DL methods and their applications in the medical domain, catering to elderly people, continue to evolve, producing better performance. As time progresses to the year 2021, more research work on disease detection in aging people using DL has been published. This paper is thus produced to offer a survey of the role of DL in aging-related issues, specifically focusing on diseases such as AMD, arthritis, cardiovascular and respiratory diseases, Alzheimer’s, and other neurodegenerative disorders while associating human genes with diseases, analysing data collected from wearable sensors for better health and lifestyle; all of the studies were published recently (2017–2021). We discussed the dynamics of predictions over health issues and diseases in the aging population, inspired by the way DL provides revolutionary insights. DL algorithms have achieved excellent performance in various predictive tasks associated with aging diseases. Henceforth, DL could also aid in the healthy aging process. The results reviewed in this work depict the promise of DL in improving the diagnosis and treatment of aging health issues.

Compared with existing related literature reviews, this article provides a scoping survey of the multiple diseases in the aging population and their diagnosis with DL with the challenges also faced in this field. Moreover, methods need to be further extended to integrate a variety of data formats of the medical domain in a DL network. To achieve a similar goal, medical imaging data, contextual data from EHRs and multiomics data can be combined leveraging DL for integration of multimodality data. Multiomics data are nowadays also widely used for disease detection and treatments. Methods adopting an integrative approach to analyse multiomics with other available medical data have the ability to address applications, such as disease prediction, biomarker identification, and deriving useful insights from medical data. DL techniques, such as autoencoder models, have the potential to integrate multiomics data with medical data for extracting representative features. Integrative analysis could potentially provide an effective way to borrow advantages from multi-level, heterogeneous medical and omics data. From the analyses of the distribution of works in the current review, the usage of CNN and its variants is used for predicting diseases involving image classification. The expansion of 2D CNN into 3D CNN is progressing, especially in the study of neurogenerative disorders in the aging population, which deals with multimodal images [17,71,81,89]. Research using DL is still evolving to achieve better performance and visibility. 

In conclusion, our review suggests that the development of novel methods making use of DL is acceptable as an objective, complementing the prediction of diseases in the aging population. To facilitate clinicians, researchers could objectively and accurately classify the diseased states using DL. The review presented valuable insights and informed the research in DL, related to the healthcare of elderly people. DL methods were used for pre-processing of medical data and in the analysis, visualization and optimization of deep neural networks in studying aging-related issues.

Research using DL is still evolving to achieve better performance. As medical data grow rapidly, research on the diagnostic classification of aging diseases is shifting towards DL models or their ensembles, integrating completely different formats of data in a DL framework. In the future, new DL modelling architectures could be designed and explored further to provide better clinical presentations for routine care of aging people.

## Figures and Tables

**Table 1:** Various kinds of DL methods.

ANN [28]	Artificial Neural Network
CNN [29]	Convolutional neural network
DNN [30]	Deep neural network
RNN [31]	Recurrent neural network
RBM [32]	Restricted Boltzmann machine
GAN [33]	Generative adversarial networks
DBM [34]	Deep Boltzmann machine
DBN [31]	Deep Belief Network
AE [31]	Auto Encoder
SAE [5]	Stacked auto-encoder
HMM [35]	Hidden Markov Model

**Table 2:** The details of scoping review.

Scoping Review Title	Role of DL in Predicting Age-Related Diseases: A Scoping Review
Review objective	To investigate the current state of DL methods in detecting age-related diseases and provide better patient care (such as early detection of age-related diseases).
Review question	How DL can help in predicting age-related diseases to understand the fundamental mechanism?
Population	Elderly human population is considered, excluding the children and youth.
Concept	The current review studies the trend in the last five years (2017–2021) of using DL, to classify predictive stages of common age-related diseases (such as AMD, Arthritis, Cardiovascular and Respiratory diseases, etc.)
Context	Use of medical imaging data, genomic data, Electronic Health Record data and data collected from wearable sensors to predict the human diseases.
Types of evidence source	Analysis of heterogenous and unstructured data to derive relevant diagnostic information aiding patients in old age.
Participants	Mostly participants with a mean age of 60–75 (nearly).

## Appendix A

**Table A1:** Application of DL in AMD.

Authors	Application	Material and Methods	Important Findings	Performance	Reference
Qi Yan et al.	In this study, both genotypes and fundus images are used to predict age-related macular degeneration (AMD) with a deep CNN.	The study used 31,262 fundus images and 52 AMD-associated genetic variants from 1,351 subjects of Age-Related Eye Disease Study over a period of 12 years. The subjects were between 55 and 80 years old at the baseline. The Inception-v3 CNN architecture was used to extract image features. Pre-trained weights were used to train the network using the ImageNet database. Three-level severity labels—no AMD, early or intermediate AMD; and late AMD were used in the study.	The study first time jointly used genotypes and fundus images in a prediction mode, enhancing the predictive performance of detecting severity of AMD.	AUC value of 0.85 (95% confidence interval 0.83–0.86).	[15]
Chuan and Yeung et al.	The study focuses on disease classification and to detect treatment-requiring retinal vascular diseases including diabetic macular edema (DME), neovascular age-related macular degeneration (named), myopic choroidal neovascularization (mCNV), branch and central retinal vein occlusion (BRVO/CRVO) in elderly people using multimodal imaging.	The study was conducted over the enrolled participants with multimodal ophthalmic imaging data from 3 hospitals in Taiwan from 2013 to 2019. EfficientNetB4 [106] was used as the convolutional neural network (CNN) as the multimodal classification model.	First study to use multimodal DL–based architecture for detecting multiple retinal vascular diseases using multiple image modalities, including retinal fundus photography, OCT, and FA/ICGA, for predicting neovascular retinal diseases. Developed model does not depend on a fixed image distribution for different modalities. The model highlighted areas with haemorrhage in retinal fundus images of subjects	High AUCs for detecting mCNV, DME, nAMD, BRVO, and CRVO were found to be 0.996, 0.995, 0.990, 0.959, and 0.988, respectively.	[67]
Peng et al.	The study deals with classification of patient-based age-related macular degeneration (AMD) severity from colour fundus photographs.	DeepSeeNet was developed consisting of three sub-networks by detecting AMD risk factors including pigmentary abnormalities for human eyes and then calculating a patient-based AMD severity score utilizing the age-Related Eye Disease Study (AREDS) simplified severity-scale. DL model was trained on 58,402 images and tested on 900 images obtained from the 4549 participants from AREDS. DeepSeeNet consists of three constituent parts that contribute to its output: (a) a sub-network, Drusen-Net (D-Net), which detects drusen in three size categories (small/none, medium, and large); (b) a sub-network, Pigment-Net (P-Net), which detects the presence or absence of pigmentary abnormalities (hypopigmentation or hyperpigmentation); and (c) a sub-network, Late AMD-Net (LA-Net), which detects the presence or absence of late AMD (neovascular AMD or central GA) DeepSeeNet is publicly available online: https://github.com/ncbi-nlp/DeepSeeNet accessed on 17 October 2021). AMD possibility increases exponentially with age: as estimated by meta-analysis at 6% at 80 years and 20% at 90 years.	The study proposed a model for better clinical decision support, mimicking the human grading process by first detecting individual risk factors of drusen and pigmentary abnormalities in each eye and then combining values from both eyes to develop an AMD score for the patient. This study shows that DeepSeeNet performed patient-based AMD severity classification with a higher level of accuracy than as predicted by group of human retinal specialists.	High AUC of >0.94 in detection of severity of AMDs.	[68]
Burlina et al.	The study highlights how DL algorithms could better characterize age-related macular degeneration from fundus images, assessing the severity of Age-Related Eye Disease.	The study utilized the AREDS data set from National Institutes of Health, including samples from 13 November 1992 to 30 November 2005, dealing with 67,401 colour fundus images from the 4613 study participants. To perform classification, deep convolutional neural networks (DCNNs) of ResNet-50 network was used. It consisted of many computational layers that performed convolutions and the related nonlinear activation operations.	DL outlines fundus and OCT image analysis, which further helps in refining retinal diagnostics in AMDs	Predicted retinal diagnostics in AMDs MAE of 3.5% to 5.3%.	[69]
Banerjee et al.	The study proposed hybrid sequential prediction model called “Deep Sequence”, integrating imaging demographic, and visual features with DL to predict the risk of exudation within AMD suffering eyes where eyes may convert from dry to wet when patients who progress to AMD. This may cause loss of vision too.	The study was conducted over the clinical trial dataset that includes 671 AMD fellow eyes with 13,954 observations of patients up to 85 years of age. It analysed longitudinal OCT imaging features and demographic information in an RNN model to predict the exudative event in eyes with AMD within 3–21 months span.	The importance of this study lies in integrating advanced imaging with sequential deep learning, RNN model, for making predictions about AMD disease progression.	Predicted exudation within AMD in the short-term (within 3 months) with 0.96 ± 0.02 AUC, and long-term (within 21 months) with 0.97 ± 0.02 AUC.	[16]

**Table A2:** Application of DL in cardiovascular and respiratory diseases.

Authors	Application	Material and Methods	Important Findings	Performance	Reference
Pengbo Zhang and Fen Xu	The study analyses and explore the application value of DL for the prediction of possible complications of coronary heart disease, and its effect on improvement of nursing and care.	DL was applied to data of 182 patients (age from 48 to 80 years old, average age: (65.27 ± 7.34) years old), collected from health records, including their previous medical history, clinical diagnosis, examination results, abnormal indicators, living habits and other information.	High-risk patients with coronary heart disease indicate relation with old age, medical history, characteristics such as lack of cognition and unhealthy lifestyle. DL Application could effectively predict the risk of related complications of heart diseases in a more accurate way.	The proposed model attained a high Accuracy of 87.5%.	[70]
Goallec et al.	Heart disease is one of the primary causes of death after age 65 and, with the world population aging. This study gain insights from DL models aiding in predicting heart age.	The study involved training of magnetic resonance videos MRI videos with 3D CNN, images with 2D CNN, time series ECG with 1D CNN over 45,000 heart MRI and electrocardiograms [ECG] from the UK Biobank within the range 45–81 years.	The study reported biomarkers, clinical phenotypes, diseases, environmental and socioeconomic biomarkers associated with accelerated heart aging. The study also highlighted the aorta, the mitral valve, and the interventricular septum as key anatomical features driving heart age prediction.	MRI-based anatomical features predicted age better than ECG-based electro-physiological features (RMSE = 2.89 ± 0.02 years vs. 6.09 ± 0.0.02 years).	[71]
Joyce D. Schroeder et al.	The study aims to predict Chronic obstructive pulmonary disease (COPD) using DL methods.	The study uses 6749 two-view chest radiograph exams (2012–2017) involving mean age as near to 60 years, also discussing COPD case of 62-year-old female. The frontal and lateral images are fed as inputs to two parallel convolutional neural networks (CNN) with pulmonary function tests (PFT) annotation.	A CNN Model trained on chest radiographs for quantitative prediction of COPD performs better than state-of-the-art algorithms of Natural Language Processing (NLP) in the field, attaining good accuracy.	AUC of 0.814 for prediction of obstructive lung disease.	[72]
Ju Gang Nam et al.	Detecting 10 common abnormalities (pneumothorax, mediastinal widening, pneumoperitoneum, nodule/mass, consolidation, pleural effusion, linear atelectasis, fibrosis, calcification and cardiomegaly) to evaluate its impact in predictive diagnostic and judging the timeliness of reporting.	The proposed approach used a ResNet34-based deep CNN over samples with mean ± SD age 57.6 ± 17.9 years on the chest radiographs.	The proposed model advanced the reporting time for critical and urgent cases, aiding better health in elderly people.	The study successfully detected 10 common abnormalities in two external validation datasets with high AUCs, ranging from 0.895 to 1.00. The training data of were curated by radiologists mostly without CT reference, intended to resemble radiologists’ performance, resulting in better results.	[73]
H. Suan-Chia Yang et al.	To predict a patient’s risk of developing lung cancer, using DL approaches	The analysis included 11,617 patients with lung cancer and 1,423,154 control patients with mean age 66 years. A total of 9261 cases of lung cancers were identified in subjects with age >= 55. CNNs have been applied to radiographic images of chest and to facilitate detection and low-dose computed tomography classification of pulmonary nodules in lungs. Xception architecture which includes a 126-layer CNN-based neural network with a moderate number of parameters, was used for feature extraction.	The study involved time-related sequential information from the medical histories to evaluate lung cancer risk in patients rather than relying on does not rely on smoking status, socioeconomic status, or BMI.	AUCs of 0.87 in patients with age ≥ 55.	[74]

**Table A3:** Application of DL in arthritis.

Authors	Application	Material and Methods	Important Findings	Performance	Reference
Kalweit et al.	The study investigated use of DL for the prediction of rheumatoid arthritis.	An adaptive recurrent neural network (AdaptiveNet), a dynamic and recurrent deep neural network architecture, designed for chronological clinical data was trained on the data collected from over 9500 patients from the Swiss Quality Management (SCQM) database.	Disease prediction was improved over patients having longer disease duration, age > 50 or having antibody positivity. Also, when compared to the ML models of linear regression, random forest and support vector machines, AdaptiveNet showed an increased performance.	Accuracy of 75.6% was achieved for disease prediction.	[75]
Leung et al.	A multitask DL model was applied over knee radiographs to accurately classify patients with high-risk osteoarthritis in both patients who underwent knee replacement (TKR) and the control patients who did not.	The proposed model used a transfer learning approach based on the ResNet34 architecture to evaluate 728 participants (with mean age, 64 years ± 8).	Accurately predicted osteoarthritis in patients who would be requiring knee replacement in 9 years.	AUC of 0.87 (95% confidence interval [CI]: 0.85, 0.90).	[76]
Norgeot et al.	Longitudinal DL models were developed for predicting disease activity of rheumatoid arthritis	A fully dense DL architecture was applied over the data of 820 patients that were extracted from the EHRs of 2 different hospitals: a university hospital (UH). rheumatology clinic (University of California, San Francisco) and a rheumatology clinic from a safety-net hospital (SNH) (Zuckerberg San Francisco General Hospital) (mean age, 57–60 ± 15 years).	Models developed for predicting rheumatoid arthritis disease from EHR data, is informative with quantifiable outcomes in the outpatient setting.	AUC of 0.91 (95% CI, 0.86–0.96)	[77]
Hirano et al.	The study predicted radiographic finger joint destruction in rheumatoid arthritis (RA).	The network of the CNN consists of two convolution layers, two pooling layers and three fully connected layers was applied over the data of 216 radiographs of 108 patients with RA, for joint evaluation. Finger joints, such as PIP, IP and MCP joints, were detected by the model and scoring scheme was used by CNN to judge the differences between joints. Radiographs in the testing dataset were used to evaluate the trained CNN model by comparing scores assigned by the model and by clinicians. Interquartile range of (53.5, 72.6) years old was taken for this study.	Processing radiographic images and determine joint destruction with the trained convolutional neural network model is promising. The authors claimed to apply CNN for the first time to detect joint destruction in RA, (particularly joint space narrowing and bone erosion of the fingers).	Accuracy reached 49.3–65.4% for Joint space narrowing and 70.6–74.1% for detecting erosion of joints.	[78]
Kwon et al.	The study aimed at developing an automated classification model for Knee osteoarthritis (KOA) based on features extracted from DL model.	Radiographic image features extracted from a DL network, namely, Inception-ResNet-v2, were further exploited using a support vector machine for KOA multi-classification.	The proposed model outperformed a common DL approach that is based on using only radiographic images as the input data.	Highest AUC of 0.93 was achieved.	[98]

**Table A4:** Application of DL in AD.

Authors	Application	Material and Methods	Important Findings	Performance	Reference
Qiao et al.	The study proposes DL classification framework with multivariate data-driven based feature extraction for automatic diagnosis of AD.	34 participants with mean age 68.64 ± 9.85 years, were taken as sample from memory outpatient clinic at the Huashan Hospital of Fudan University. A total of 34 participants with mean age 65.55 ± 8.98 years, were invited by public advertisement to take part in the study. The proposed method was based on a three-level hierarchical partner matching independent component analysis (3LHPM-ICA) and Granger causality (GC) to determine effective connectivity features playing role in AD diagnosis.	Identified brain features that can serve as important biomarkers for AD.	Accuracy of 95.59% in diagnosing AD	[80]
Qureshi et al.	The study performed automatic assessment of dementia severity using a DL framework applied to resting-state functional magnetic resonance imaging (rs-fMRI) data.	The demographics included the participants with mean age of 73. The 3D-CNN-based DL classification framework is used in this study to assess dementia.	The research supported automatic classification of AD into two groups of disease severity (very mild and mild vs. moderate and severe) enabling important contributions for clinical practice.	Accuracy of >90% was achieved for the disease classification.	[81]
Choi and Jin et al.	The study aims to develop an automatic image interpretation system based on a deep convolutional neural network (CNN) to predict future cognitive decline in mild cognitive impairment (MCI) patients using flurodeoxyglucose and florbetapir positron emission tomography (PET)	Deep CNN was trained using 3-dimensional PET volumes of AD. The data used in this study included subjects recruited in Alzheimer’s Disease Neuroimaging Initiative-II (ADNI-2) with available baseline data on FDG and AV-45 PET (http://adni.loni.usc.edu, accessed on 17 October 2021) with a mean age of 73 years.	Importance of DL as a practical tool for developing predictive neuroimaging biomarker.	Accuracy of 84.2% to predict cognitive decline in AD.	[82]
Ding et al.	A DL algorithm is used for an early prediction tool for Alzheimer’s disease providing therapeutic intervention by using biochemical and imaging tests.	PET imaging studies from 1002 patients, from Alzheimer’s Disease Neuroimaging Initiative (ADNI)-1, ADNI2, and ADNI-GO (Grand Opportunities) studies was considered. The average age of the patients was 76 years (range, 55–93 years) for men and 75 years (range, 55–96 years) for women. Convolutional neural network architecture, Inception v3, was used in this study stacking over 11 modules where each module consists of pooling layers and convolutional filters with rectified linear units as activation function over the two-dimensional images of 16 horizontal sections of the brain.	A DL algorithm developed for successful early prediction of Alzheimer’s disease by using fluorine 18 fluorodeoxyglucose PET of the brain.	AUC of 98% for predicting AD.	[83]
Lu et al.	The study is aimed at identifying the stage of Alzheimer’s Disease (AD) patients through the use of mobility data and DL models.	The study applied a state-of-the-art architecture deep convolutional neural network, Inception-ResNet-V2, to pre-train brain images involving elderly people of age > 65.	Modelling to classify AD, showed outstanding characteristics as a progression biomarker. Interestingly, the study involved weighted brain structural image and information on participant sex.	Accuracy of AD classification found to be >90%.	[84]
Huang et al.	The research proposes a practical brain imaging-based AD diagnostic classifier using DL/transfer learning on MRI dataset of unprecedented size and diversity.	The data were obtained from the Alzheimer’s Disease Neuroimaging Initiative (ADNI) database. The study chose 2861 T1-MR images, including AD subjects with a mean age of 76.13 ± 7.50. The study proposed a multi-modality CNN-based classifier for AD diagnosis and prognosis.	The work distinguishes between AD or potential AD patients from cognitively unimpaired (labelled as CN) subjects accurately and automatically using medical images to facilitate a fast-preclinical diagnosis.	AUC of 92.01% to differentiate between AD and Controls.	[85]

**Table A5:** Some more applications of DL dealing with aging-related issues.

Authors	Application	Material and Methods	Important Findings	Performance	Reference
Titano et al.	The study applied a novel DL architecture to a clinically heterogeneous set of three-dimensional optical coherence tomography (OCT) scans from patients referred to a major eye, London, United Kingdom.	3D U-Net architecture was used to determine referral recommendation to clinicians for disease prediction involving OCT scans of majority people of age > 50, on a range of sight-threatening retinal diseases. Data was collected about raw retinal OCT scans around the macula). Deep segmentation network was trained with manually segmented OCT scans to determine tissue map images. Deep classification network, was then trained over the tissue maps with diagnoses of disease with predicted diagnosis probabilities providing recommendations to the clinicians.	The study presented a novel framework that analyses clinical 3D OCT scans and makes referral suggestions to a standard comparable to clinical experts.	AUC of >0.96 with a taxonomy of different diagnostic labels suggested to clinicians.	[89]
Betancur et al.	The study applied DL for prediction of obstructive disease from myocardial perfusion imaging (MPI). Obstructive disease was defined as ≥70% narrowing of coronary arteries.	The study comprised of 1638 patients from 2008 to 2015, at 9 national and international (Canada, Switzerland, Israel) sites with mean age of 65.2 ± 11.0 in obstructive disease. The feature maps comprised of 3 fully connected layers transformed the image features into the final scores.	The potential of DL for prediction of obstructive diseases from MPI, as compared with the standard quantitative analysis of total perfusion deficit (TPD).	AUC disease prediction by DL was higher (0.80 per patient) than current standard method (0.78 per patient).	[90]
Weiya Shi et al.	The study applied DL model based on quantitative computed tomography (CT) images to predict the severity of current global pandemic COVID-19.	196 patients with confirmed COVID-19 those were enrolled from 20 January to 10 February 2020, Shanghai Public Health Clinical Centre, Fudan University, China, were considered for analysis. CT images help in studying prognostic and diagnostic characteristics of COVID-19, the quantification of these with DL is useful to predict he severity of disease. The median age of the severe group considered in this study was 65 years. Methodology utilized the V-shaped neural network (V-Net) which is a convolutional neural network (CNN) and combined it with transfer learning to segment the infected regions on images.	DL over CT model containing radiological features serve as an efficient tool for prediction of COVID-19 severity. CT suggests disease progression and it was found that with other age-related diseases, CT has prognostic value for predicting mortality.	A high AUC of 0.900 was achieved for predicting COVID severity.	[91]
Varadarajan et al.	DL is applied to extract novel features such as refractive error from retinal fundus imaging in a novel way.	Retinal fundus images used in this study were obtained from the UK Biobank and the Age-Related Eye Disease Study (AREDS) clinical trials. Refractive error is one of the most common causes of visual impairment in age related issues worldwide. A DL model residual network (ResNet) consisting of three residual blocks, an attention layer to learn the most predictive eye features, and two fully connected layers was trained to predict the refractive error from fundus images. Poor quality images were excluded to be fed into DL model.	Successfully found features that predict refractive error in retinal fundus in patients.	MAE of 0.91 D (95% CI: 0.89–0.93) was obtained on the AREDS clinical validation data set.	[92]
Nguyen et al.	The study applied DL model combined with generalized linear model improve the prediction of the onset of type 2 diabetes mellitus (T2DM)	The dataset consists of identified electronic health records for 9948 patients, of which 1904 have been diagnosed with T2DM of age-range up to 84 years.	Using DL over EHRs aids better in predicting the onset of diabetes and useful in improving the quality and efficiency of medical care	AUC of 84% is achieved by proposed method.	[19]
Jonsson et al.	Aging has a significant structural impact on the brain that correlates with decreased mental and physical fitness. The current study focusses on determining brain age to predict neurodegenerative diseases from a T1-weighted MRI.	The proposed method uses a 3D CNN trained on MRIs to predict brain age. CNN is validated on 104 images from the IXI dataset left out during training (validation set) and tested on 12,395 images from the UK Biobank dataset. CNN derives on five residual blocks, each followed by a max pooling layer of stride 2 × 2 × 2 and kernel size 3 × 3 × 3, and one fully connected block.	T1-weighted MRIs are useful to predict brain age associating them with neuro degenerative diseases and achieved promising results.	MAE of 3.996 was achieved with CNN.	[17]
Fabris et al.	The study was aimed at predicting whether human genes are associated with several age-related diseases at the same time using Deep Neural Network (DNN) methods.	DNN with three fully connected layers followed by an output layer to predict whether each s gene is associated with age-related diseases (27 classes of diseases). Human protein coding genes were obtained from NCBI BioMart v. 87, and age-related disease gene associations were the subset of the Genetic Association Database.	Popular genes were identified that are strongly linked to aging-related diseases. Use of DNN improved the predictive performance.	AUC > 85% was achieved with DNN for predicting age-related diseases.	[48]
Basheer et al.	The study aims towards early prediction of dementia using CNN (convolutional neural networks), a DL method is over neuroimaging data	150 subjects aging from 60 to 96 years old. The study fed images in a type of image retrieval system, using the CNN method and the proposed technique of using CapNet. The proposed model uses the modified capsule network technique for classifying the dementia groups into demented and non-demented category.	Age is the most dependent feature for Dementia. The proposed model can handle hierarchical modelling problems and is computationally more efficient with better accuracy as compared with the other models.	Accuracy of 92.39% for predicting dementia.	[93]
Cheon et al.	The study proposes use of deep neural networks to detect stroke patient mortality using medical service and health behaviour data	Subjects collected from 2013 to 2016 by the Korea Centres for Disease Control and Prevention with mean age of 66 years. The study proposed DNN/scaled principal component analysis (PCA) to automatically generate features from the data and identify risk factors for stroke. Feed-forward neural networks were trained using a standard backpropagation.	Early detection of patients at high risk of stroke who would need additional attention before disease exacerbation.	AUC of 83.48% to predict stroke patient mortality.	[94]

**Table A6:** Application of DL in predicting other aging-related issues using sensor-based data.

Authors	Application	Material and Methods	Important Findings	Performance	Reference
Papagiannaki et al.	Use of DL in physiological monitoring of older people using wearable sensors for improving their health status and quality of life.	The study involved convolutional neural network architectures (CNNs) in recognizing movement patterns of older people. Three CNN layers were used and of which each convolutional layer is followed by a normalization layer and a rectified linear unit (ReLU) activation unit. The recordings were obtained from older people aged 70–92 who participated in the FrailSafe project available online: https://frailsafe-project.eu/ (accessed on 17 October 2021).	The study proposed DL-driven activity recognition scheme for older people involving feature extraction with classification demonstrating advantages of DL techniques.	More than 80% Accuracy over different smart sensor locations in physiological monitoring of old people.	[95]
J. Prince et al.	The study proposes detection and assessment of Parkinson’s disease (PD) using wearable sensors, highlighting the suitability of DL over the collected data.	Analyses was conducted over the sample of 1815 participants over the age of 45 years.	The study indicated similarity between classification of manually extracted feature set, and the features learnt by a CNN in diagonisis of PD. The study also indicated the suitability of DL for assessing PD when large population is available	DL approaches capable of PD disease classification, performed better than traditional methods.	[86]
Mishkhal et al.	The purpose of this study is to identify daily activities that consider a key to falling cases for older adults at care homes.	A deep activity CNNet was used to determine features recorded from daily activities sensors data and to associate them with highest falling risk activities in testing data. People aged 66–86 years, participated in the experiments.	DL techniques aid in effectively assessing fall risk based on wearable sensors looking for a way to keep the elder people’s lives safe and better.	Achieved an Accuracy of >96% in different settings.	[87]
Rajdeep Kumar Nath et al.	To distinguish between stressed and non-stressed state in older adults using wearable technologies and application of DL to improving the quality of life in aging. Chronic stress is one of the risk factors in contributing to the development of Alzheimer’s Disease (AD) also in older people.	A finger-tip sensor was used during the Trier Social Stress Test, to reliably induce stress in humans (within social setting) to record signals. DL model of recurrent neural network using Long Short-Term Memory (LSTM) was applied over the collected data to classify older people into stressed or non-stressed state. Data of 19 healthy older adults (11 female and 8 male) with mean age 73.15 ± 5.79 were used for this study.	DL-based LSTM classifier attained better performance than traditional ML models in distinguishing between stressed and non-stressed state. An efficient system for unobtrusive wearable device system for physiological stress detection in older adults was devised aiding in prevention of cognitive decline in older adults.	High AUC score of 0.90 was achieved with DL using LSTM.	[88]
Lu et al.	The study is aimed at identifying the stage of Alzheimer’s Disease (AD) patients through the use of mobility data and DL models.	The study applied a state-of-the-art architecture deep convolutional neural network, Inception-ResNet-V2, to pre-train brain images involving elderly people of age > 65.	Modelling to classify AD, showed outstanding characteristics as a progression biomarker. Interestingly, the study involved weighted brain structural image and information on participant sex.	Accuracy of AD classification found to be >90%.	[84]
Huang et al.	The research proposes a practical brain imaging-based AD diagnostic classifier using DL/transfer learning on MRI dataset of unprecedented size and diversity.	The data were obtained from the Alzheimer’s Disease Neuroimaging Initiative (ADNI) database. The study chose 2861 T1-MR images, including AD subjects with a mean age of 76.13 ± 7.50. The study proposed a multi-modality CNN-based classifier for AD diagnosis and prognosis.	The work distinguishes between AD or potential AD patients from cognitively unimpaired (labelled as CN) subjects accurately and automatically using medical images to facilitate a fast-preclinical diagnosis.	AUC of 92.01% to differentiate between AD and Controls.	[85]
